# A Surrogate Artificial Neural Network Model for Estimating the Fatigue Life of Steel Components Based on Finite Element Simulations

**DOI:** 10.3390/ma18122756

**Published:** 2025-06-12

**Authors:** Ela Marković, Tea Marohnić, Robert Basan

**Affiliations:** University of Rijeka, Faculty of Engineering, Vukovarska 58, 51000 Rijeka, Croatia; ela.markovic@student.uniri.hr (E.M.); robert.basan@riteh.uniri.hr (R.B.)

**Keywords:** surface-hardened steel materials, finite element analysis, artificial neural networks, fatigue life

## Abstract

A surrogate artificial neural network (ANN) model trained on the data generated from a computational finite element-based (FE-based) model is developed. The developed ANN model enables the estimation of the fatigue life (number of load cycles to failure) of component-like specimens with stress concentrators. Using the developed model, the component-specific *S*-*N* curves can be generated with an accuracy comparable to that of the computational FE-based model. The investigation covered through- and surface-hardened steel components with different numbers and types of stress concentrators. The basis for data generation is the parametrized computational FE-based model, which enables the determination of the stress–strain response and the calculation of the fatigue life of examined components under cyclic loading conditions. The computational FE-based model can be adjusted to include components with different geometries and heat treatment conditions. The computational FE-based model incorporates nonlinear material behavior to provide a more accurate representation of the component’s behavior, which results in higher computational costs. In contrast, the developed ANN model provides a quicker and more efficient way to assess the fatigue life of both through- and surface-hardened components, overcoming these limitations.

## 1. Introduction

Most machine elements used in demanding fields, like the aerospace, automotive, or heavy machinery industries, are subjected to intense static and dynamic loads, leading to possibly significant, locally concentrated stresses. These stress concentrations significantly impact the component’s overall functionality, potentially leading to the failure of the entire component. Unlike the failure caused by static loads, fatigue failure can happen abruptly, without the presence of significant plastic deformation, making the detection of component damage or predicting failure more difficult [1].

Regions of structural steel components expected to experience concentrated stresses are often selectively heat-treated to improve surface hardness and strength while aiming to preserve the ductility of the core [2]. This approach is commonly applied to machine elements such as metal gear teeth. Surface hardening results in gradual variations in material properties, including hardness, strength, and residual stresses, from the higher-strength surface to the lower-strength core, which can generally improve the component’s fatigue strength depending on the geometry and loading conditions.

One of the main prerequisites for an accurate assessment of the mechanical behavior and fatigue life of surface-hardened components is understanding their material properties and mechanical response under various conditions, either through experimental investigation or computational methods.

### 1.1. Fatigue Testing and Assessment

The ability of a material to resist fatigue failure is generally assessed by fatigue life, defined as the number of load cycles to failure, *N*_f_, that a standardized test specimen can endure before it fails. Failure can refer to either the final fracture or the initiation of a macroscopic crack [1]. In this study, focus is placed on the initiation of fatigue cracks, so *N*_f_ represents the number of load cycles required for crack initiation. Fatigue testing is always necessary when a component is subjected to cyclic loadings, particularly for different heat-treated components. Fatigue testing of surface-hardened components presents additional difficulties compared to homogeneous (through-hardened) components. This is because the tested specimens need to represent the materials of such components, and for that, at least two groups of different heat-treated specimens are needed, with one group representing the material at the surface and the other representing the material at the core. In contrast, through-hardened components only require a single type of specimens. This demonstrates that fatigue testing of components with different heat treatments is both time-consuming and costly, which is why, in the early stages of product development and engineering design process, the assessment of the load-bearing capacity and durability of components usually involves computer simulations with complex modeling techniques combined with fatigue life assessment methods, reducing the number of experiments that need to be conducted [3,4]. Computational models are mostly based on finite element analysis (FEA) and are capable of more accurately capturing the mechanical behaviors of components with complex material properties as opposed to traditional analytical models, and they can lead to more efficient engineering design processes, extending the component’s lifetime and decreasing material usage by reducing the need for extensive experimental investigations.

To support such simulations, various fatigue life assessment methods have been developed to estimate the durability of components under cyclic loading. The most commonly used concepts include the stress-based, strain-based, and fracture mechanics approaches. Stress- and strain-based methods assume fatigue failure occurs upon crack initiation [5], while the fracture mechanics approach focuses on modeling crack propagation and is not considered in this work. Stress-based approaches are particularly suitable for high-cycle fatigue scenarios, where low nominal stress levels result in predominantly elastic material behavior [6].

Unlike stress-based approaches, which can use, for example, nominal stresses or local stresses at the notch, the strain–life approach can account for local plastic strain in regions where fatigue cracks initiate. This makes it particularly suitable for components with stress concentrators, where local plasticity plays a significant role in fatigue crack initiation [6]. This approach requires modeling the elasto-plastic behavior at critical locations and is typically described using the Basquin–Coffin–Manson (BCM) equation, which combines elastic and plastic strain components. However, the BCM equation assumes zero mean stress and therefore requires modifications to handle more general loading conditions. Among the extended models, Smith et al. [7] proposed that fatigue life is best governed by the product of maximum tensile stress and total strain amplitude, where the maximum tensile stress can be defined as the sum of stress amplitude and mean stress. Consequently, this approach can be categorized as stress/strain-life approaches, and it accounts for the effect of mean stress by considering the maximum stress during one cycle. This model, also called the Smith, Watson, and Topper (SWT) model [7], provides a reliable estimation of the fatigue life and mean stress effects in the high-cycle fatigue regime, though it tends to be conservative in predicting low-cycle fatigue behavior [8,9,10].

### 1.2. Literature Review on Surrogate Models for Material Behavior Estimation

While computational models remain the primary approach for modeling mechanical behavior in most engineering components, the advancement of machine learning techniques presents new opportunities to improve or even fully replace conventional numerical methods [11,12,13]. In this context, various machine learning approaches, including artificial neural networks (ANNs), have been successfully used to approximate material behavior and replace finite element simulations in various applications, showing comparable accuracy with significantly reduced computational cost. ANN models are particularly useful in optimization scenarios, where a large number of function evaluations are required in each iteration. Replacing finite element simulations with surrogate models in such cases can significantly reduce computational time. Building on these developments, this work explores the use of ANN-based surrogate models for predicting the fatigue life of surface-hardened steel components with stress concentrators as an alternative to the computational model based on finite element analysis.

One of the areas of application of ANN-based surrogate models is the aviation industry, where it is crucial to estimate the maximum stresses on highly loaded parts during operation. In order to avoid slower and more complex simulations using the finite element method, Hovanec et al. [11] applied an approach using artificial neural networks. They developed a method for predicting the maximal stress during operation on aircraft torque tubes. They varied input variables such as the brake temperature, outside temperature, and landing speed. Similarly, Spodniak et al. [12] developed an innovative methodology for predicting temperature fields on jet engine turbine blades based on the integration of the finite element method and artificial neural networks.

Ashank et al. [14] developed a convolutional neural network model trained on finite element simulation data to design composite microstructures for targeted elastic properties. The model takes microstructure images as input and, once trained, predicts the composite materials’ homogenized macroscopic properties for any given composite microstructure. Also researching composite materials, Shokrollahi et al. [15] developed an advanced predictive model based on a convolutional neural network (CNN) to map stress fields within microstructure images of composite components. They developed a 2D finite element model that enabled the generation of a variety of composite geometries and used this data to train a CNN surrogate model. The trained CNN effectively predicts stress field maps with high accuracy for composite microstructures with varying numbers and layouts of fibers.

Greve et al. [16] employed a surrogate machine learning approach to replace finite element simulations in the topology design of structural components, such as those used in vehicles. Their FE model simulated an academic case of a flat metal sheet with holes under tensile loading up to fracture, representing a typical crash scenario. Two surrogate models were developed and trained on data from these FE simulations, both demonstrating strong predictive capabilities for the post-necking indicator, which served as an output to identify structural cracks. Umbrello et al. [17] developed a hybrid FEM-ANN approach for predicting residual stress profiles after the hard machining process, demonstrating that ANNs can generalize FEA results to provide quick predictions of residual stress distributions. Their method not only accurately predicts the effects of cutting tool geometry, material properties, and machining parameters but also offers an inverse strategy to determine the required process conditions for a desired residual stress profile. Additional examples of using surrogate artificial neural networks to replace FE simulations are discussed in the review paper by Kumar et al. [18], with a focus on models evaluating the structural integrity of corroded pipelines. This review covers extensive ongoing research in this field and presents promising results, highlighting the potential use of surrogate models in similar applications.

In addition to the presented general engineering applications, several studies have explored the use of artificial neural networks specifically for fatigue life prediction, which is the focus of this work. Dresia et al. [19] demonstrated the potential of applying artificial neural networks for fatigue life prediction in complex engineering systems. By training an ANN on a large dataset generated through a finite element analysis of rocket engine combustion chambers, they achieved reliable fatigue life estimates with significantly reduced computational time. Xu et al. [20] developed machine learning models trained on the results from finite element simulations and experimental investigations to predict the fatigue life of the structural components made of Ti_2_AlNb-based alloys. The models demonstrated very good predictive capability when compared to experiments. Feng et al. [21] developed a real-time fatigue life prediction model using a feedforward neural network (FNN) trained on data points generated via extended finite element simulations with randomly sampled stochastic parameters. Structural responses were used as input features. The FNN outperformed other investigated machine models showing the most reliable predictions. Chen et al. [22] proposed a hybrid approach combining finite element modeling and artificial neural networks to predict fatigue life in wafer-level chip-scale packages. Finite element simulations were used to analyze the influence of structural parameters on maximum creep strain under thermal fatigue. The resulting data was used for ANN training for strain and fatigue life prediction, achieving a strong predictive performance and reducing computation time.

The literature review on the applications of artificial neural networks shows their potential as more efficient and accurate alternatives to finite element simulations, especially for structural components with complex material properties. Successful implementations of surrogate machine learning models for predicting mechanical behavior and/or optimizing designs across various engineering fields are demonstrated. The findings of these studies, especially those addressing component fatigue life prediction, support the use of surrogate ANN models as a promising method for estimating the fatigue life of surface-hardened engineering components, which is the focus of this work.

This study presents an ANN surrogate model for the estimation of the fatigue life of through- and surface-hardened steel components with stress concentrators as a replacement for the computational model based on FEA. A parametric computational model based on finite element analysis is initially developed and presented in the following section, providing a foundation for developing an artificial neural network model.

## 2. An Overview of the Underlying FE-Based Computational Model

A computational model based on finite element analysis that enables the estimation of the expected fatigue life (number of load cycles to failure) for through- and surface-hardened steel components with stress concentrators is developed as a part of ongoing research and is partially published in [23]. This model serves as the foundation for generating the data used to train the surrogate artificial neural network model proposed in this study. In this section, a brief overview and necessary details of the developed FE-based computational model are provided.

The FE-based computational model consists of two key parts, a finite element analysis of a component, which enables the determination of its stress and strain amplitudes, and a computational part, in which the number of load cycles to failure are calculated. The FE model can capture the hardness distribution in both through- or surface-hardened steel materials. For surface-hardened steels, it captures the gradual transition in material strength from the high-strength surface to the lower-strength core, as well as the variation in residual stresses. The computational model is designed for through- and surface-hardened steel materials, particularly steels intended for case hardening or quenching and tempering.

Approximation methods are used to define individual cyclic stress–strain material curves, where each of them is related to the corresponding hardness value of the material. Generally, cyclic stress–strain curves are defined by connecting the tips of the stabilized hysteresis loops obtained during cyclic loading tests and represent the relationship between stress and strain amplitude for a material subjected to repeated loading and unloading cycles. The stabilized cyclic stress–strain curves were constructed using Ramberg–Osgood parameters (*K*′ and *n*′) [24] estimated directly from hardness values, based on the expressions proposed by Lopez and Fatemi [25], with the implementation methodology detailed in [26]. These continuous curves were then discretized using the MVD algorithm (also detailed in [26]), enabling their implementation into the FE model as multilinear isotropic material models. Based on the values of obtained stress and strain amplitudes, the number of load cycles to failure were estimated using a Smith–Watson–Topper strain-based approach with mean stress correction since it was shown to provide good estimates in high- and low-cycle regimes and is still widely used [8,10,27,28].

More specifically, the uniaxial tensile–compressive loading conditions were applied to the investigated component in the FE model, reflecting the stress-controlled loading scenarios typically used in the fatigue testing of specimens to generate stress–number of cycles (*S*-*N*) diagrams. When assessing the effects of stress concentrators or inhomogeneous stress distributions on fatigue life, *S*-*N* curves can be generated to reflect these conditions, making them component-specific and representative of the component’s behavior [1]. Since the model is parametrized, multiple consecutive analyses for specific geometry combinations under different loading conditions can be simulated, enabling the construction of a characteristic *S*-*N* curve for a given component.

**Figure 1:**
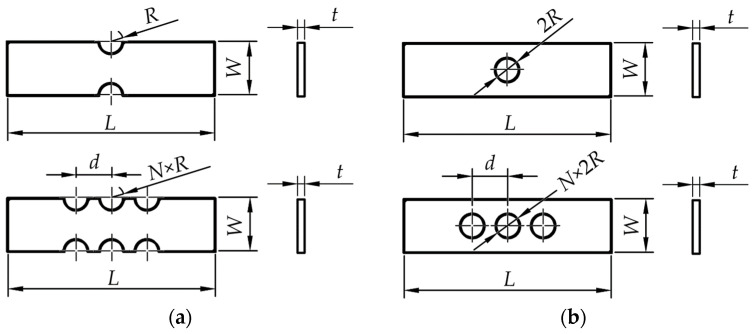
Parametrized component geometry with rectangular cross-section with thickness *t*. (**a**) Component with semicircular opposite single or multiple notches. (**b**) Component with central single or multiple holes.

The component under consideration is a thin plate with two different types of stress concentrators, opposite single notches and circular holes. Additionally, the study also considers multiple opposite notches and holes in such elements. Examples of component geometries are illustrated in Figure 1, using examples of components with two and six opposite notches and single and three circular holes. The spacing between the stress concentrators (*d*), when multiple are present, is defined as *d* = 2·*R* + 7 mm, ensuring a consistent 7 mm of material between two stress concentrators in all cases based on preliminary empirical results.

Given that the component structure is thin and flat, with the length and width being much larger than its thickness and loaded by in-plane forces, the model can be further simplified to a 2D representation of an actual component by applying the plane stress condition [29,30,31]. Under plane stress conditions, it is assumed that the stress in the direction perpendicular to the plane (the thickness direction) is negligible. Consequently, the normal stress in the thickness direction and the shear stresses acting in the thickness direction are considered zero. Only the in-plane stresses are taken into account, and they do not depend on thickness under this assumption [29].

**Figure 2:**
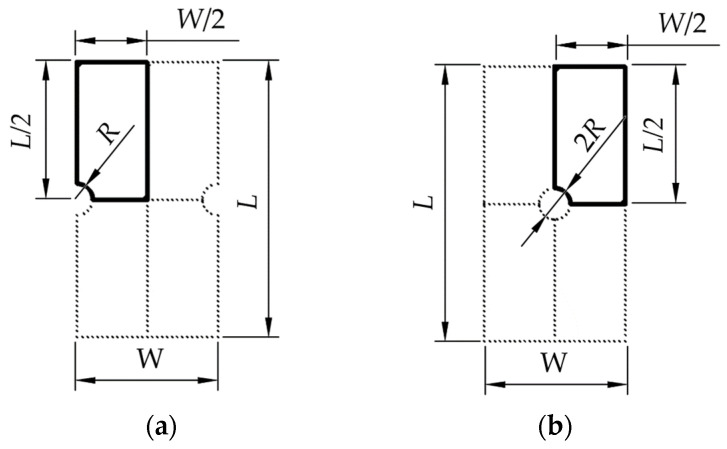
The entire component under investigation shown in 2D, divided with symmetry lines emphasizing a quarter model with parametrized variables in the FEA. (**a**) The component with a semicircular opposite single notch; (**b**) the component with a central hole.

The plasticity-related nonlinear steel material behavior is included in the finite element analysis, which significantly increases the computational time required for the solution. To address this, the component is simplified to a 2D representation of the actual component and reduced to a quarter model using symmetry conditions. The component geometry is first divided into four quarters along both horizontal and vertical lines of symmetry, as shown using examples of components with opposite notches and a central hole in Figure 2. This approach simplifies the analysis and saves time since geometry consists of four times fewer nodes for which displacement and, consequently, strains and stresses need to be computed numerically.

Symmetry conditions are then applied to these cut lines to account for the symmetrical behavior of the specimen. Uniaxial loading conditions are applied to the component in the FE model, reflecting stress-controlled loading scenarios typically used in the fatigue testing of specimens. For this approach, nominal or reference stress needs to be defined. When dealing with components with stress concentrators, two types of nominal stresses can be defined, gross nominal stress (*S*_g_) and net nominal stress (*S*_n_). In this case, gross nominal stress is used to define the loading condition in the FE model since this corresponds to the stress that would be applied in a real test scenario. Gross nominal stress is defined at a location far enough from the stress concentrator and is calculated as the equivalent force required to achieve the desired nominal stress level during the simulation divided by the gross cross-sectional area. On the other hand, net nominal stress is defined by dividing the equivalent force by the net cross-sectional area, which is obtained by subtracting the stress concentrator from the gross cross-section. For the construction of *S*-*N* curves, the net nominal stress of the component is used.

**Figure 3:**
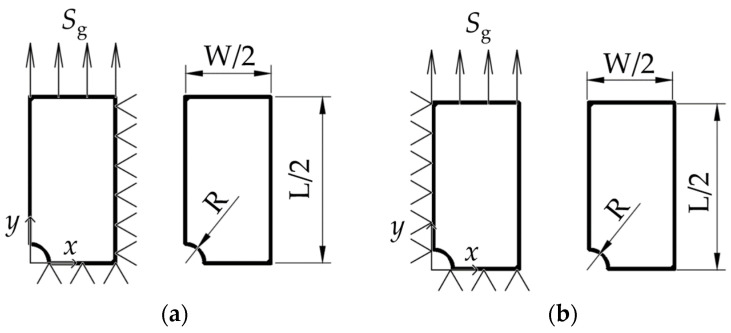
Boundary and symmetry conditions for modeled quarter specimen geometry and parametrized variables including length, width, and radius. (**a**) Component with opposite notches; (**b**) component with single central hole.

The boundary and symmetry conditions applied to the quarter model of the component along with the parametrized variables for the specimen geometry are illustrated in Figure 3 with the example of a component with two opposing notches and a single central hole. For the components with more stress concentrators, the same symmetry and boundary conditions are applied. The model is symmetric regarding both the horizontal and vertical mid-planes, allowing for reduction to a quarter geometry.

The component is modeled and parametrized in APDL (Ansys^®^ Mechanical APDL), with geometrical parameters including the half-width, half-length, the radius of notches or holes, the number of notches or holes, and the spacing between them. Other relevant properties of the FE model not specifically mentioned here are summarized in Table 1.

After defining the geometric variables and material parameters, such as the surface and core hardness, the FE model provides the nodal distribution of stress and strain amplitudes across the model geometry. Before calculating fatigue life, the stress ratio *R,* defined as the ratio of minimum to maximum stress in a loading cycle, needs to be specified. In this study, *R* = 0 (zero-to-tension loading) is assumed, meaning the mean stress equals the stress amplitude. After this, the number of load cycles to failure across all nodes of the model is determined using the Smith–Watson–Topper [7] strain-based approach, mentioned in Section 1, with the mean stress correction, which is applicable for a wide range of metal materials:

(1) $$\varepsilon_{a}\left(\sigma_{a}+\sigma_{m}\right)=\left({\sigma^{\prime }}_{f}^{2}/E\right)\;{\cdot \left(2N_{f}\right)}^{2b}+{\sigma_{f}}^{\prime }{\varepsilon_{f}}^{\prime }{\left(2N_{f}\right)}^{b+c}.$$

where *ε*_a_ is the total strain amplitude, while *σ*_a_ and *σ*_m_ denote the stress amplitude and mean stress amplitude, respectively, obtained from the FE model. The fatigue parameters from that expression, *b* and *c*, are assigned constant values of −0.09 and −0.56, respectively, while *σ*_f_′ and *ε*_f_′ are determined for each hardness value using Equations (2) and (3), as proposed by the hardness method of Roessle and Fatemi [32], which has been widely applied in the literature [4,33]. The hardness method was developed for steels with a medium to high carbon content. Since this study focuses on through- and surface-hardened materials, particularly steels used for quenching and tempering or intended for case hardening, the hardness method and corresponding parameter values are applicable.

(2) $$\sigma_{f}^{\prime }=4.25HB+225,$$

(3) $$\varepsilon_{f}^{\prime }=\left(0.32HB^{2}-487HB+191,000\right)/E.$$

In Equations (2) and (3), *HB* denotes the Brinell value of hardness at a specific location in the component, and *E* is the Young’s modulus, assigned a value of 2.06 × 10^5^ MPa, which is commonly used for steel.

The presented computational FE-based model enables the determination of mechanical response and the calculation of fatigue life for through- and surface-hardened components. It incorporates nonlinear material behavior and nodal-level material variations, which ensure a more accurate representation of the component’s behavior but significantly increase simulation times. The iterative nature of FEA and the need to solve partial differential equations make it time-consuming to fine-tune model parameters, even for minor geometric or material changes. For example, modifying the surface hardness requires redefining the hardness distribution, updating all component nodes, adjusting cyclic material parameters, and reassociating them within the material model. Achieving a balance between accuracy and computational efficiency is essential as higher accuracy demands finer meshes and more complex material definitions, further increasing the computing time. Even with simplifications such as 2D modeling, plane stress assumptions, and symmetry conditions, simulations remain computationally intensive, especially for complex geometries or material states. These high demands limit the number of design variations that can be explored within a reasonable timeframe.

As a result, a surrogate ANN model as a substitute for the FE-based computational model is developed and presented in the following section. The developed surrogate ANN model is trained on the data generated from the finite element model and enables the estimation of the fatigue life of through- and surface-hardened components with stress concentrators across various loading conditions, geometries, and material conditions.

## 3. Materials and Methods

The introduced parametrized numerical model enables the simulation of components with stress concentrators with various geometries and material conditions. As already stated, a balance between computational time and accuracy is necessary, as detailed finite element models that capture complex material behavior provide greater accuracy but increase computing time. To address these challenges, a new methodology using artificial neural networks is developed for faster assessment of the mechanical behavior and, consequently, fatigue life of components with complex material and geometrical properties, in this case, components with stress concentrators. In the next section, the workflow of the methodology that employs artificial neural networks to develop a surrogate model, replacing the finite element model for surface-hardened component-like specimens, is presented.

### 3.1. The Workflow of the Surrogate ANN Model

**Figure 4:**
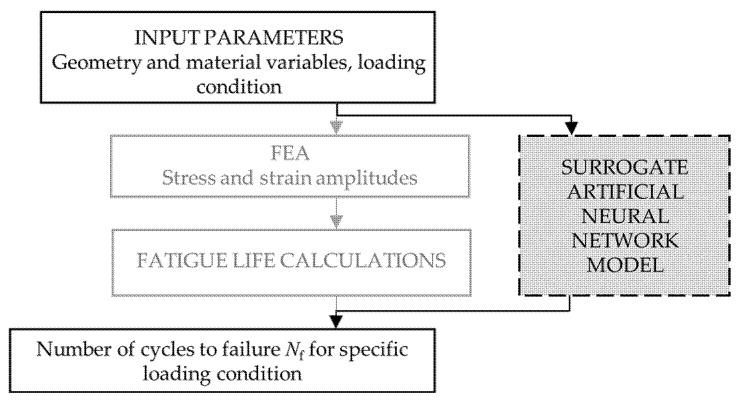
Workflow of computational model for fatigue life assessment with highlighted surrogate artificial neural network.

The surrogate artificial neural network model, which integrates FEA and fatigue life calculations and was developed to replace the computational model presented in the previous section, is illustrated in the workflow chart presented in Figure 4.

The computational model, which includes both FE analysis and fatigue life calculation, is replaced by a surrogate ANN model capable of estimating the fatigue life of the component for specific component geometry and corresponding loading conditions. While this approach significantly increases computational efficiency, it only calculates the fatigue life of the component rather than providing the fatigue life distribution across all nodes. However, it is not necessary to have information about the fatigue life of every node since the shortest fatigue life of a specific node determines the overall component fatigue life, which makes this method sufficient.

**Figure 5:**
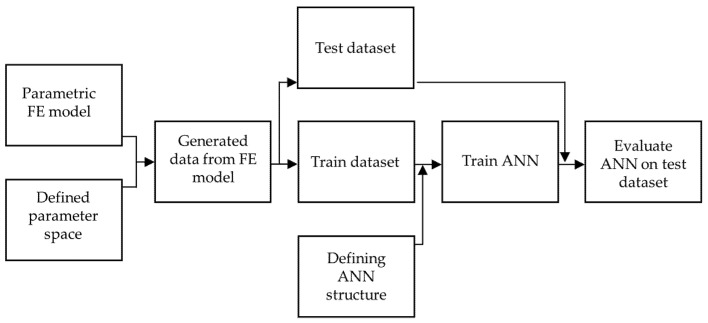
Workflow of developing and training artificial neural network surrogate model to be used instead of finite element model.

The general workflow, i.e., the main steps in the development of the surrogate model, is illustrated in Figure 5. To develop the artificial neural network surrogate model, a training dataset must be provided. This dataset is created by performing multiple finite element analyses with varying input parameters, including different geometries and material properties, as well as different loading conditions. Following this, the structure of the artificial neural network is defined, and the ANN is then trained on the FE-generated data. Finally, the ANN is evaluated using a test dataset that was not included in the training process.

The following sections outline the key steps of this workflow, starting from defining the parameter space to evaluating the ANN on the test dataset.

### 3.2. Input Variable Parameter Space for Forming Training and Test Datasets

The data used to train the ANN model was generated from the parametric computational FE-based model. For this, the space of parameters was defined, which includes key input parameters (geometry and material variables) that influence the FE model. The range of parameters has to be such that it ensures that the surrogate model generalizes different scenarios well. After this, multiple FE simulations following nodal fatigue life calculations (using Equation (1)) are performed by varying the input parameters within the defined space. Output variables for each geometry and loading combination are recorded, in this case, component fatigue life, *N*_f_. The results from each FE analysis form the dataset used to train the ANN model.

**Figure 6:**
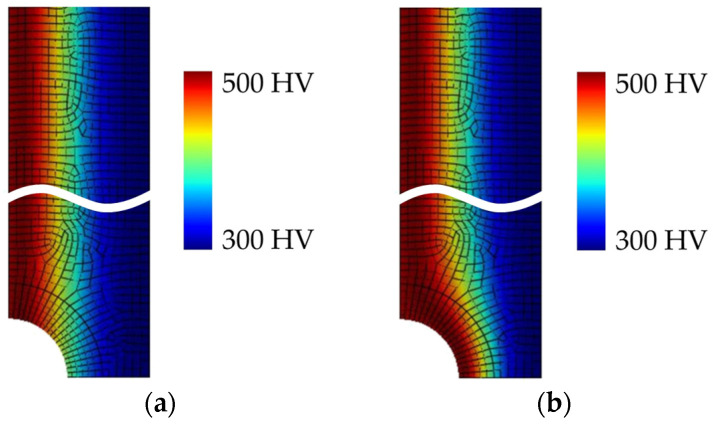
Illustration of two scenarios of surface-hardened components with notches, using quarter model representation from FE model. (**a**) Notches introduced after heat treatment (surface hardening); (**b**) notches introduced before heat treatment (surface hardening).

Parametrized variables in the developed computational FE-based model can be separated into geometry and material variables. Geometry variables include the half-length (*L*/2), half-width (*W*/2), stress concentration definition (opposite notches or a central hole), the radius (*R*), and the number of stress concentrators (*N*). Material variables include information about the heat treatment of the component, indicating whether it is surface-hardened (defined by different values of surface and core hardness, *HV*_s_ and *HV*_c_, respectively) or through-hardened. Various combinations of surface and core hardness are employed to simulate different possible heat treatment scenarios, such as quenching followed by tempering at different temperatures, resulting in varying hardness distributions. Additionally, two scenarios are considered: one involving the introduction of notches (by machining) before surface hardening and the other occurring after surface hardening. The second scenario represents a more complicated case since the surface of the stress concentrator is not equally hardened and the obtained hardness at the stress concentrator depends on its radius and corresponding hardness profile. Figure 6 illustrates both scenarios for a specific geometry and material combination: a thin component with a total length of 120 mm, a width of 24 mm, and a notch radius of 5 mm, with surface and core hardness values of 550 HV and 300 HV, respectively, while assuming plane stress conditions.

The length of the component was adjusted for each combination to accommodate the intended number of stress concentrators. Generally, for thin planar specimens with stress concentrators, the length has minimal influence on the local stress and strain distribution as long as it extends well beyond the area of interest, which follows Saint-Venant’s principle. However, if the specimen is too short, boundary conditions may interfere with the stress and strain distributions around the concentrator. To avoid this, a minimum component half-length of 80 mm was used and increased when more stress concentrators were present, ensuring that enough material remained outside this region for stress and strain distributions to become nearly uniform.

The possible values of variables, whose combination forms the space of parameters for training the ANN, are presented in Table 2. All the variables listed in the table are physically relevant to the problem as they significantly influence the final component fatigue life. Therefore, all were involved in training the ANN model to ensure no important information was lost.

The number of different values that each parameter can take is as follows:

- Half-width—11 levels;
- Radius—11 levels;
- Number of stress concentrators—8 levels;
- Types of stress concentrators—2 levels;
- Hardened or non-hardened notch—2 levels;
- Surface-hardened (8 levels) or through-hardened (4 levels)—12 levels combined.

In statistical terms, this setup resembles a factorial experiment, and the complete set of combinations across all parameters would amount to 46,464 (11 × 11 × 8 × 2 × 2 × 12) combinations in a full factorial design space. Although this scenario involves six parameters with discrete levels, it is important to note that not all combinations of these factors are possible. After applying constraints, only 20,992 valid combinations are possible. The constraints include

- The exclusion of combinations where the radius exceeds 55% of the half-width of the component, chosen empirically;
- The exclusion of combinations involving a hardened notch with homogeneous properties or a central hole.

When a hardened notch is selected, only eight combinations of surface-hardened properties are possible. On the other hand, if a non-hardened notch is chosen, there are either eight combinations for surface-hardened or four for through-hardened properties (12 combined).

Given the time required for each FE simulation, analyzing every combination for just a single loading condition was not feasible, which is why design of experiments (DOE) methods were considered. While approaches such as Taguchi design [34] are often used to identify influential factors with fewer experiments, in this case, all factors were assumed to have equal importance for the resulting stress, strain, and, consequently, fatigue life, so a random subset of the full factorial space was selected, consisting of 400 combinations (approximately 2%). Each geometry combination was additionally subjected to ten loading conditions, resulting in a total of 4000 datasets (400 × 10 loading cases) to train the artificial neural network.

The test dataset used to evaluate the ANN was generated using the same principle as that outlined for the train dataset, but it did not originate from the same parameter space, ensuring no overlap with the training data. To assess the network’s generalization ability, which is defined as the ability of the network to perform well on unseen inputs [35], different parameter combinations, including the widths, radii, and hardness values not included in the training set, were utilized for testing. The space of parameters used for generating the test dataset is listed in Table 3. This approach ensures that the model is evaluated on data that represents scenarios not directly encountered during training, providing a strong assessment of its ability to generalize to new, unseen conditions.

Following the approach used to develop the training set and considering only valid parameter combinations, along with the same restrictions, the total possible combinations amount to 17,920. A fractional factorial design is similarly applied here, with the test dataset being 30% of the training dataset’s size, resulting in a total of 120 datasets. With ten loading conditions per dataset, this amounts to 1200 test datasets. If the artificial neural network performs well on the test dataset, it can be concluded that the model generalizes effectively, accurately handling values that fall between the predefined ones.

### 3.3. Generating Data to Train the Surrogate ANN Model

For visualization purposes, 3 of the 400 combinations of input variables used to generate the dataset to train the artificial neural network model are presented in Table 4. The full dataset with different combinations of geometry and material input variables for generating training and testing datasets are provided in the Supplementary Material (Tables S1 and S2) accompanying this study. Each of these 400 combinations was subjected to ten loading conditions, resulting in a total of 4000 finite element analyses that were conducted.

After performing 4000 analyses using the computational FE-based model described in Section 3, the training dataset for the surrogate ANN model was generated. Table 5 presents 6 of the 4000 dataset entries, corresponding to the three combinations of input variables listed in Table 4. In terms of loading conditions, each examined component is subjected to ten different uniaxial loading conditions, not exceeding a maximum total strain amplitude of 3%. Two out of ten of these loading conditions are presented for each combination number. The output variables from FE analyses needed for the subsequent calculation of fatigue life include strain and stress amplitudes, as well as mean stress amplitudes. All examined scenarios assume a zero-to-tension loading with a stress ratio of *R* = 0, in which case the mean stress, also needed for the calculation of fatigue life, is equal to the stress amplitude. The output variable used to train the surrogate ANN model is the number of load cycles to failure, as shown in the table. Its values span over a wide range, from a couple of cycles to 10^7^ cycles (with a maximum of 10^9^ cycles in the full dataset). The full training and testing datasets are provided in the Supplementary Material (Tables S3 and S4) accompanying the study. Due to this extensive range, the variable is transformed logarithmically and shown in Table 5.

### 3.4. Training the Surrogate ANN Model

This section outlines the structure of the ANN model and explains the training process, detailing the specific parameters used, which are then applied to train the network. A key aspect of the training process is the preprocessing of the input and output variables, which considers the transformation of the data into a suitable form. Preprocessing steps for the output variable are explained, followed by a discussion of the transformations applied to the input variables and the regularization techniques used to improve model performance and prevent overfitting. After this, variables that determine the neural network structure and impact the performance, called hyperparameters, are selected. Lastly, the growth method is used to determine the optimal number of neurons in the hidden layer.

#### 3.4.1. Defining the Structure of the ANN Model

**Figure 7:**
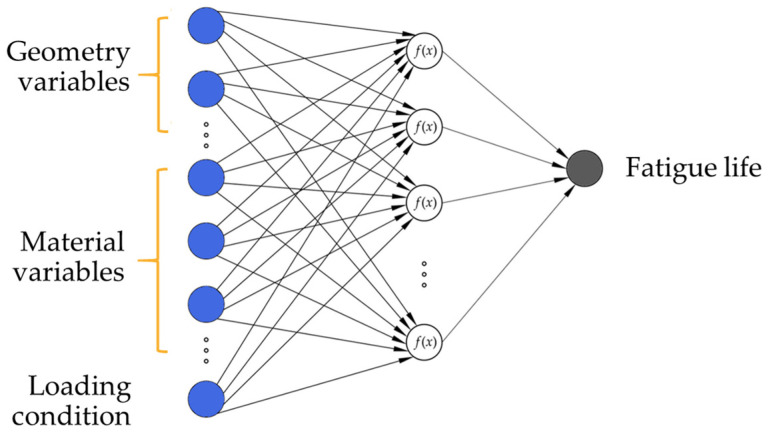
The architecture of the fully connected artificial neural network model with one output, predicting the fatigue life of examined components.

Artificial neural networks are composed of three types of layers, an input layer with one neuron per input variable, one or more hidden layers containing multiple neurons, and an output layer with one neuron per output variable. For the given problem, i.e., the prediction of the component fatigue life of examined components, a two-layer multilayer perceptron with a linear transfer function in the output layer was developed. The neurons in the hidden layer are fully connected to the neurons in the input and the neurons in the output layer. Using the linear transfer function is appropriate in this case as the target variable (fatigue life) is continuous and unbounded. Figure 7 shows a schematic representation of two-layered ANNs employed in this study.

#### 3.4.2. Preprocessing of Input and Target Variables

The input and target variables require careful preprocessing to ensure that they effectively contribute to the learning process. As stated in Section 4.2, not all combinations of input parameters were physically possible, which is why the training and test datasets were created with the specified constraints. For example, surface-hardened components with notches may have both non-hardened and hardened notch configurations, which naturally appear more frequently in the dataset compared to components with holes. Because of this, the natural data imbalance of input variables was not artificially adjusted.

Typically, input variables should be on a similar scale so that no single variable disproportionately influences the model. To achieve this, appropriate scaling techniques were applied. In this study, two types of input variables are present: categorical and continuous variables. Categorical variables, which include the type of stress concentrator (notch or hole) and whether the notch is hardened or unhardened, were preprocessed using one-hot encoding. This method converts categorical variables into numerical ones by creating binary columns for each category, for example, assigning a value of 1 for a notch and 0 for a hole, making them suitable to use when training the ANN model. Continuous variables, such as the radius of the stress concentrator, the half-width of the component, surface hardness, core hardness, and the loading condition, were preprocessed by data transformation. Specifically, min–max normalization was employed, which performs a linear transformation on the input variables and rescales each variable to fall within the range of 0 to 1 [36]. This approach keeps the relationships among data points while ensuring that all continuous variables contribute equally to the training process. Normalization can prevent inputs with larger values from dominating the learning process and can improve the accuracy of the network [36].

**Figure 8:**
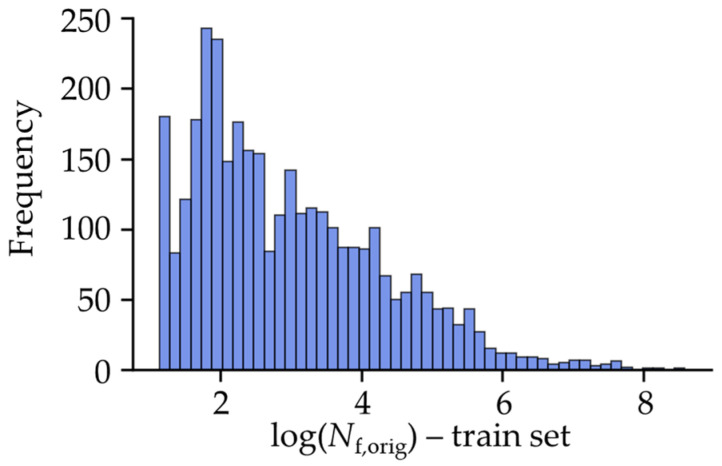
The histogram of the logarithmically transformed target variable (fatigue life, *N*_f_).

The fatigue life target variable spans several orders of magnitude, ranging from less than 100 to a maximum of 10^9^ cycles. While components that survive beyond a given number of cycles are often treated as run-outs in experimental fatigue analysis, this case is not applicable to this model. The full training and testing datasets, including the target variable, can be seen in the Supplementary Material (Tables S3 and S4). It is common practice to present *S*-*N* diagrams on a logarithmic scale. Generally, variables with such wide-ranging scales are typically unsuitable for effective ANN model training and can negatively impact model performance. Additionally, the fatigue life target variable has a concentration of values at the lower end of the fatigue life spectrum, resulting in a non-uniform distribution. This is because in the low-cycle fatigue regime, components exhibit significant plastic deformation where the relationship between the stresses and strains is no longer linear. It is expected that this complexity makes the ANN’s learning process more challenging in the LCF range compared to the HCF range. This skewness in the data can significantly (negatively) affect model performance. To address these issues, scaling is necessary. In this case, since the fatigue life is commonly represented on a logarithmic scale, a logarithmic transformation is considered appropriate. The logarithmic transformation additionally helps to reduce the impact of extreme values, spreading the accumulated points more evenly. Therefore, the fatigue life target variable was logarithmically transformed and presented in a histogram in Figure 8. The fatigue life target variable, even after scaling, remains skewed towards the lower end of the value range but this distribution was found to be acceptable for model training.

#### 3.4.3. Model Configuration

The selection of key model parameters, including the optimization algorithm, activation function, and loss function, plays a crucial role in the model’s performance. In addition, hyperparameters, which are predetermined variables that are not adjusted by the learning algorithm, significantly influence the overall effectiveness of the model [35]. Hyperparameters should be selected using a separate validation dataset that is not observed by the training algorithm to prevent overfitting the network [35]. Overfitting is a common issue in artificial neural network modeling, where the model may memorize the training data and thus perform poorly on new, unseen data, failing to generalize effectively. Because of this, several regularization strategies were included in the network. Regularization refers to adjustments made to a learning algorithm aimed at reducing generalization error without affecting training error [35]. In this study, L1, L2, and elastic net regularization methods were explored. Additionally, early stopping was implemented to prevent overfitting.

Preliminary experiments with several combinations of model parameters and hyperparameters were conducted to assess the performance of the network on the validation dataset, which included 15% randomly chosen observations of the training dataset. The empirically chosen network configurations, hyperparameters, and regularization techniques, along with explanations of their selection and underlying principles, are detailed in the following paragraphs.

The optimization algorithm adjusts the model’s parameters to optimize the objective (loss) function, typically by minimizing it. A variety of optimization algorithms are available, each with strengths and trade-offs. Among these, adaptive learning rate algorithms, such as RMSProp or Adam, have demonstrated fairly robust performance for training artificial neural networks. RMSProp, for example, was empirically shown to be a reliable choice for deep learning tasks [35]. Similarly, Adam, an optimization algorithm that combines the advantages of RMSProp and momentum, is widely regarded for its robustness and efficiency. One of Adam’s key benefits is its ability to adapt the learning rate for each parameter dynamically, which often leads to faster convergence and reduced sensitivity to hyperparameter tuning [35,37]. Adam is also recognized for achieving impressive results in terms of test set accuracy [37]. While there is no single best algorithm for every problem, in this study, the Adam optimization algorithm was chosen for this ANN model, with the learning rate hyperparameter (α) set to 0.001, after it was empirically observed to yield better results than RMSProp.

Activation functions are essential for computing hidden layer values in neural networks. The rectified linear unit (ReLU), defined as *g*(*z*) = max{0,*z*}, is commonly used due to its simplicity and efficient optimization [35]. ReLU can cause issues during training because it outputs zero for negative values which can make many units output zero, limiting learning. To address this, Leaky ReLU introduces a small negative slope for negative values, with α typically set at 0.01. Before the introduction of rectified linear units, most neural networks used the hyperbolic tangent activation function: *g*(*z*) = tanh(*z*) [35]. For this problem, Leaky ReLU with hyperparameter α set at 0.01 slightly outperformed ReLU and tanh.

The objective of training a neural network is to minimize (or, in some cases, maximize) an objective function, often referred to as the loss function. The loss function quantifies the difference between the model’s predicted outputs and the true target values. For this study, mean squared error (*MSE*) [38] was selected as the loss function based on empirical observations as it consistently outperformed alternatives such as the mean absolute error (*MAE*) during model development.

The batch size is a hyperparameter that defines the number of training examples the learning algorithm processes before updating the internal model parameters. It is common when training neural networks to use more than one but fewer than all the training examples [35]. In these cases, each mini-batch is used to compute the gradient and update the weights. This means that in one epoch, weights are updated the amount of time as there are batches [35]. For this problem, smaller batch sizes (16 and 32) led to better model generalization, with 32 chosen as the final batch size.

Early stopping is a widely used regularization technique that addresses overfitting by monitoring the validation error. During training, while the training error steadily decreases, the validation error may begin to increase, indicating overfitting. To address this, the model’s parameters are saved each time the validation error improves [35]. If no improvement occurs after a set number of iterations (patience = 10 in this case), the parameters corresponding to the lowest validation error are used. This technique reduces computational costs and is applied in this work.

L1 or L2 (or a combination of both, elastic net) regularization adds penalties to the loss function based on the absolute value of weights to improve model generalization and prevent overfitting [35]. L1 regularization (lasso) forces some of the weights to become zero, which is why it can sometimes be viewed as a form of feature selection and can cause sparse solutions [38]. L2 regularization (ridge) prevents the weights from becoming too large but does not set them to zero, which results in the model having smaller weights and allowing for model complexity to be reduced. The combined approach combining the advantages of both L1 and L2 regularization is called elastic net. L2 regularization achieved the highest performance among different regularization techniques (L1, elastic net, and without regularization were also tested) with an alpha value of *λ* = 0.001.

The final network settings with defined hyperparameters are presented in Table 6.

After determining optimal hyperparameters, the final number of neurons in the hidden layer was determined with the growth method, starting from one neuron in one hidden layer.

#### 3.4.4. The Determination of the Number of Neurons in the Hidden Layer

The number of neurons in the neural network was determined using a growth method to systematically determine the optimal number of neurons in the hidden layer, starting from one neuron. In this approach, the number of neurons in the hidden layer gradually increased from 1 to 60, and the *R*-squared score as well as validation loss determined with mean squared error were recorded on the validation dataset. This process was repeated three times to assess the stability of the approach by calculating the standard deviation for each neuron count.

**Figure 9:**
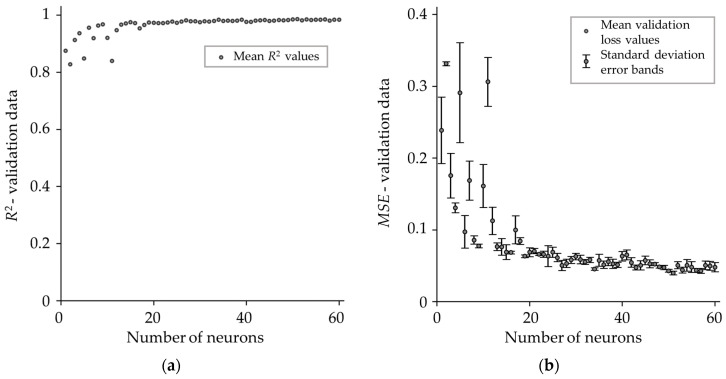
Growth method for determining optimal number of neurons in hidden layer. (**a**) *R*-squared score for different numbers of neurons in single hidden layer ANN; (**b**) validation loss for different number of neurons in single hidden layer ANN.

Figure 9 presents the mean *R*-squared score (Figure 9a) and mean validation loss (Figure 9b) for different numbers of neurons in a single hidden layer, obtained from three independent analyses, along with error bars. As can be seen, both the *R*-squared score and validation loss *MSE* improved significantly with an increasing number of neurons, stabilizing between 20 and 30 neurons, where the model achieved high accuracy. The figures also show standard deviation error bands, with the standard deviation of *MSE* values starting at approximately 0.05 for a single neuron and decreasing to around 0.006 at 20 neurons, after which it remained relatively stable.

After further analysis, 23 neurons were selected as the optimal number since this value minimizes validation loss and achieves a very high *R*-squared value. Adding more neurons risks overfitting without significant performance improvement.

## 4. Results and Discussion

After developing the artificial neural network for predicting component fatigue life, its performance must be assessed using an independent test dataset. The evaluation, detailed in the following subsections, includes quantitative metrics, *N*_f,pred_-*N*_f,orig_ scatter diagrams comparing predicted and target fatigue life values, and an example of a component *S*-*N* curve generated by the ANN model.

### 4.1. Evaluation of Predictive Performance Using Key Metrics

The predictive performance of the developed model was assessed across the entire dataset. Additionally, to provide more context, the fatigue life estimates on the test set were grouped into several relevant categories. Specifically, the data is separated based on fatigue life ranges and different heat treatments. Fatigue is commonly categorized into high-cycle fatigue (HCF) and low-cycle fatigue (LCF), with the transition typically occurring somewhere between 10,000 and 100,000 cycles, though this limit is not strictly defined. In this study, the threshold between them is set at 10⁴ cycles to failure, as a practical midpoint within the commonly referenced range, particularly for steels subjected to case hardening or quenching and tempering. Additionally, heat treatment is separated into two categories, distinguishing between through-hardened components, which have uniform hardness and strength throughout, and surface-hardened components, with different values of hardness and strength from the surface towards the core.

For each of the mentioned categories, the performing metrics are presented in Table 7. Since a small gap between training and test errors is also an important factor contributing to the determination of the generalization capability of the model, metrics are presented on the training dataset as well.

As the original values of fatigue life vary significantly in scale, ranging from less than 100 to a maximum of 10^9^ cycles, the measures presented in Table 7 are based on target (FE-based) values and predicted values in logarithmic scale, without converting them to the original scale. To convert the values to the original scale, the predicted and target values would need to be exponentiated, but for the purpose of model evaluation, the logarithmic scale is sufficient and more meaningful given the wide range of values. Evaluation in the original scale is performed in the next section and presented in the *N*_f,pred_-*N*_f,orig_ scatter diagrams, where more appropriate measures for scale variation are used.

The metrics presented in Table 7 include the coefficient of determination (*R*^2^), mean absolute error (*MAE*), mean absolute percentage error (*MAPE*), mean squared error (*MSE)*, and root mean squared error (*RMSE*).

*R*^2^ is a statistical measure that indicates the proportion of variance in the dependent variable that is predictable from the independent variables in regression models. Values range from 0 to 1, with 1 indicating perfect prediction. *MAE* measures the average of the absolute differences between predicted and target values. Since the errors are not squared as with other measures, it gives equal weights to all errors. It shows the average error magnitude without considering the direction and is in the scale of the original target variable, in this case, logarithmic values of fatigue life, *N*_f_. The *MAPE* can be defined as the percentage equivalent of the *MAE* as it measures the average of the absolute percentage errors between predicted and target values. It is useful for understanding the relative size of errors. Also, the individual target values *y*_i_ must not be zero to avoid division by zero, which is not a concern in the case of predicting fatigue life, as all values are larger than zero. *MSE* quantifies the average squared difference between the predicted and target values, providing an overall measure of error magnitude. The *RMSE* is the square root of the *MSE* and expresses the error in the same units as the target variable, making it more interpretable.

When comparing the model’s performance on the training and test datasets, it is evident that the model consistently provides better estimates for the training dataset than for the test dataset, as expected, while still providing generally accurate predictions overall. This is supported by high *R*^2^ values for both datasets (0.952 for training and 0.935 for testing) and relatively low *MAE* values (0.201 for training and 0.255 for testing). A more intuitive measure in this context is the *MAPE*, expressed as a percentage, which gives values of 7.618% and 9.739% for the training and test datasets, respectively, indicating relatively low average errors. Additionally, the *MSE* and *RMSE* values are relatively low (0.085 and 0.291 for the training set; 0.129 and 0.359 for the test set), indicating the model’s good performance. Better performance on the training dataset is expected since the model was trained using this data. Also, the small differences in performance between the training and test datasets suggest that the model generalizes well and is not overfitting. It is worth noting that the test dataset was generated from a separate parameter space than the training dataset, which further emphasizes the robustness of the model’s predictive capabilities on unseen data.

Regarding the performance of the network on different groups of data, including the fatigue life ranges and different heat treatments, conclusions can be drawn based on the metrics presented in the table.

When comparing fatigue life ranges, the LCF range shows better predictive capability, as indicated by the majority of the performance metrics with specific values listed in the table. The *MAPE* is the only metric that shows an advantage in the HCF range, with a value of 6.664% compared to 10.746% in the LCF range. This discrepancy can be attributed to the smaller values of fatigue life in the LCF range, where even small absolute errors can lead to larger relative (percentage) errors. In contrast, the larger values in the HCF range tend to result in smaller percentage errors, even though the absolute errors (*MAE*) are larger. Notably, the *R*^2^ value is higher for the test dataset when all the data is analyzed together (0.935) compared to when it is divided into the LCF (0.833) and HCF ranges (0.734). This suggests that the two ranges may complement each other, resulting in improved overall model performance. This highlights the need for an additional metric to evaluate the data differently, as demonstrated in Section 4.2 using the original scale of actual and predicted values of fatigue life. In conclusion, it appears that the difference in predictive capability over the range of fatigue values is not overly prominent, and the model generalizes well across both ranges, although it does show a slight advantage in the LCF range.

Upon examining both through-hardened and surface-hardened cases, it is clear that the model exhibits better predictive capability when estimating through-hardened components, with an *R*^2^ value of 0.975 and an *MAPE* of 5.582%, compared to the surface-hardened components, which have an *R*^2^ value of 0.924 and an *MAPE* of 11.315%. Other metrics are also in favor of the through-hardened cases. This difference is expected, as determining stresses and strains from FEA is more straightforward for through-hardened components. As discussed in the introduction, modeling surface-hardened materials and acquiring solutions through FEA presents additional challenge due to the variation in hardness and strength across the component. The through-hardened component is modeled with a single stress–strain cyclic curve representing the behavior across the entire component, which is not the case for the surface-hardened component being described with multiple stress–strain cyclic curves depending on the hardness and strength at certain positions. Despite the challenges, the predictive capability of the ANN model for surface-hardened components is strong and the results are considered satisfactory.

After examining all results, the developed ANN model demonstrates strong predictive capability across all tested cases, including different fatigue life ranges and components with varying heat treatments. These findings suggest that the model can be effectively applied to a wide range of fatigue life scenarios, providing reliable predictions across a range of cases within the boundaries of the data it was trained on.

**Figure 10:**
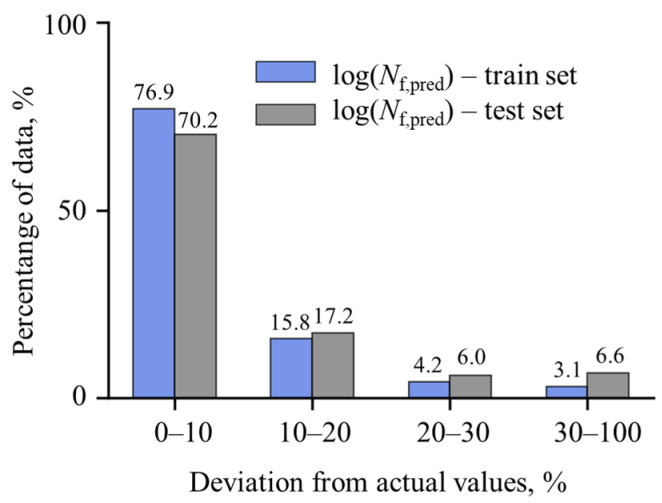
Percentage of fatigue life values estimated by developed ANN that deviate in selected percentage ranges compared to target (obtained with FE-based computational model) values on test dataset.

To further evaluate the predictive accuracy of the artificial neural network in estimating the fatigue life of components with stress concentrators, Figure 10 presents a comparison of the percentage of estimates falling within specific deviation ranges relative to target results (obtained using the FE-based computational model) for both the training and test datasets.

The results indicate that over 75% of the fatigue life estimates for the training data fall within ±10% deviation, with the test dataset showing a slightly lower value of approximately 70%. Similarly, over 90% of the training data estimates deviate by up to ±20%, compared to 87.4% for the test data. The relatively small difference in predictive performance between the training and test datasets, combined with high predictive accuracy, indicates that the ANN model achieves strong generalization without overfitting.

### 4.2. Evaluation of Predictive Performance Through N_f,pred_-N_f,orig_ Diagrams

The evaluation conducted so far has considered the logarithmically transformed target fatigue life variable, as obtained from the ANN prediction. Although the model was trained on the entire fatigue life range, the differences in performance when the data was analyzed separately for the low- and high-cycle fatigue ranges were apparent. The *R*^2^ value was higher for the test dataset when all data was considered together compared to when it was split into the two ranges. This suggests that while the model performs well overall, further metrics are needed to understand the model’s behavior within each range. Since the focus of this study is estimating the fatigue life of components, the output variable is transformed back to represent the number of load cycles to failure in order to construct scatter *N*_f,pred_*-N*_f,orig_ diagrams. A more intuitive understanding of the predictive performance of the model is given by presenting additional metrics on the original scale.

**Figure 11:**
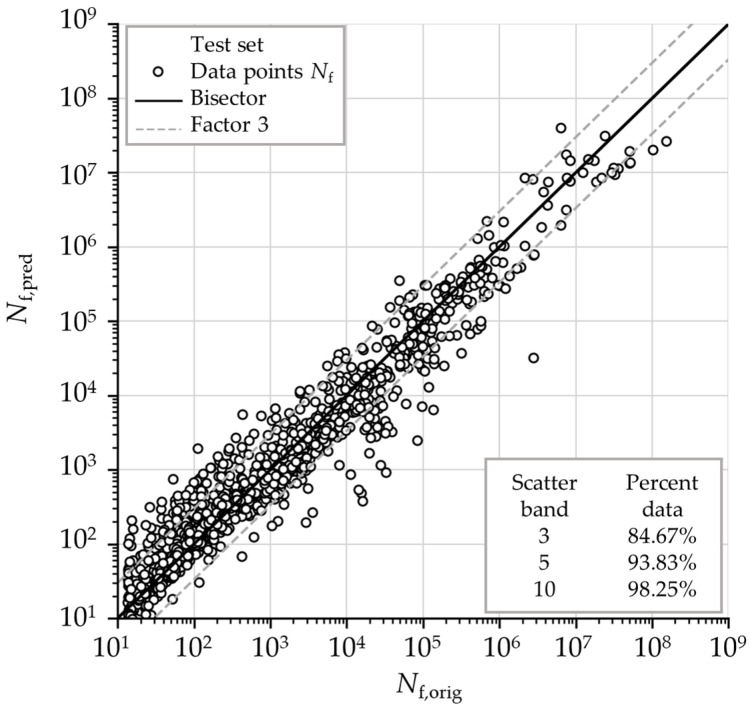
Scatter diagram of predicted vs. target values of component fatigue lives on unseen (test) data.

Figure 11 presents a scatter diagram illustrating the relationship between target values (FE-based) and the predicted values of component fatigue life derived from the developed ANN on the test dataset. The figure also includes the ideal correlation line and scatter band lines for a life factor of 3 as well as additional percentages of predicted fatigue life data points falling inside the band range with fatigue life factors of 3, 5, and 10. It is observed that 84.67% of the data points fall within the constructed range (band with a fatigue life factor of 3) and that the alignment with the bisector is relatively good. When using a band with a fatigue life factor of 10, over 98% of the data points fall within this range, indicating good generalization.

**Figure 12:**
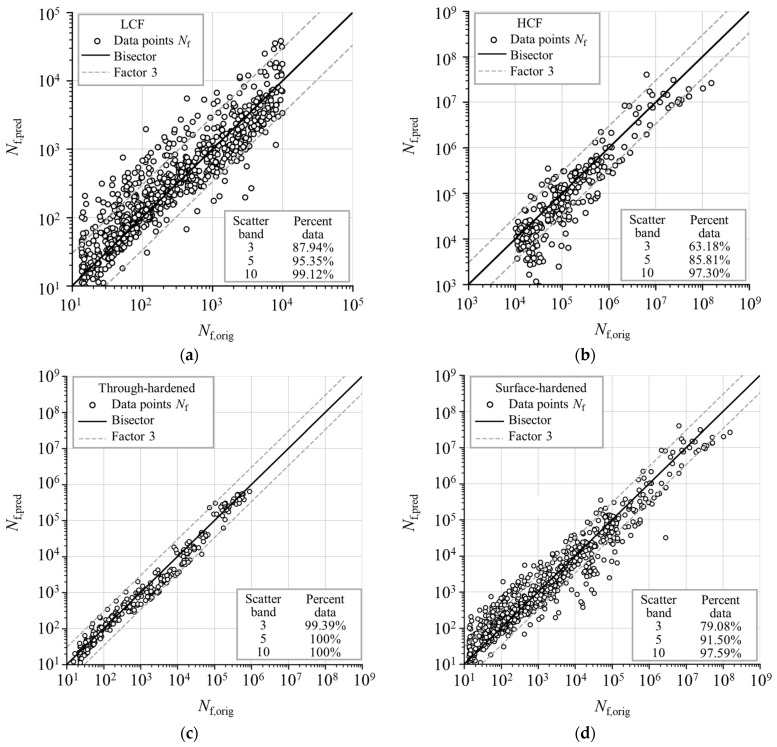
Scatter diagrams of predicted vs. target values of number of load cycles to failure on test dataset, with data separated by number of load cycles and heat treatment. (**a**) Low cycle fatigue range—up to 10^4^ cycles; (**b**) high cycle fatigue range—from 10^4^ cycles. (**c**) Through-hardened components; (**d**) surface-hardened components.

To further analyze the predictive performance of the developed model, the same categorization of the data based on fatigue life ranges, and different heat treatments, as mentioned earlier, are used. Based on this categorization, four *N*_f,pred_-*N*_f,orig_ diagrams are constructed and presented in Figure 12, with bands representing the deviation of original data points with a fatigue life factor of 3 as well as additional percentages of predicted fatigue life data points falling inside the band range with fatigue life factors of 3, 5, and 10.

The upper diagrams in Figure 12 show the *N*_f,pred_-*N*_f,orig_ diagrams for two fatigue life ranges. Fatigue failure in the LCF range is characterized by localized plastic deformation, which adds complexity to finite element analysis due to the need for nonlinear material models as the relationship between stresses and strains is no longer linear, as already stated. As a result, the ANN model may encounter greater difficulty learning the data points within this range. Nevertheless, it can be observed that the cloud of data points predicted by the ANN in the low-cycle fatigue range exhibits less scatter compared to that in the high-cycle fatigue range. Additionally, there are fewer data points in the HCF range, which may contribute to the greater scatter of the cloud of data points. Both diagrams align closely with the bisector, indicating relatively good predictive accuracy without significant deviation from the expected trend.

The percentage of predicted data points falling within the range defined by a fatigue life factor of 3 is higher in the LCF range (87.94%) than in the HCF range (63.18%). These observations indicate that the ANN model performs more consistently within the LCF range, while the HCF range shows greater variability in the predicted data points. In the LCF range, 10.29% of the estimated data points fall on the non-conservative side outside of the fatigue life factor of 3 bands, while 1.77% fall on the conservative side outside of the fatigue life factor of 3 bands. Conversely, in the HCF range, 5.4% of the estimates are outside the fatigue life factor of 3 bands on the non-conservative side, while 31.42% are on the conservative side. To conclude, the developed ANN model shows better predictive capability in the LCF range than in the HCF range, with more conservative estimates in the HCF range.

The lower diagrams in Figure 12 show the different component heat treatment conditions, representing through- and surface-hardened components. First, it is important to note that predicting the surface-hardened components with stress concentrators is a more difficult task than just homogenous (through-hardened) components. This can also be directly observed in the scatter diagrams. Specifically, 99.39% of the data points of through-hardened components fall inside the range with a fatigue life factor of 3, and when observing a range with a factor of 5 or higher, all data points fall inside of it. On the scatter diagram representing the surface-hardened components, 79.08% of the data points fall inside the range with a factor of 3, and more than 90% fall inside the range with a factor of 5. Here, 9.75% of the data points are predicted outside the fatigue life factor of 3 on the conservative side, and 11.17% on the non-conservative side, indicating a relatively balanced distribution around the bisector. It can be concluded that while estimating the fatigue life of surface-hardened components is more challenging, the model still provides rather reliable estimates.

### 4.3. Example of Component-Specific S-N Diagram

**Figure 13:**
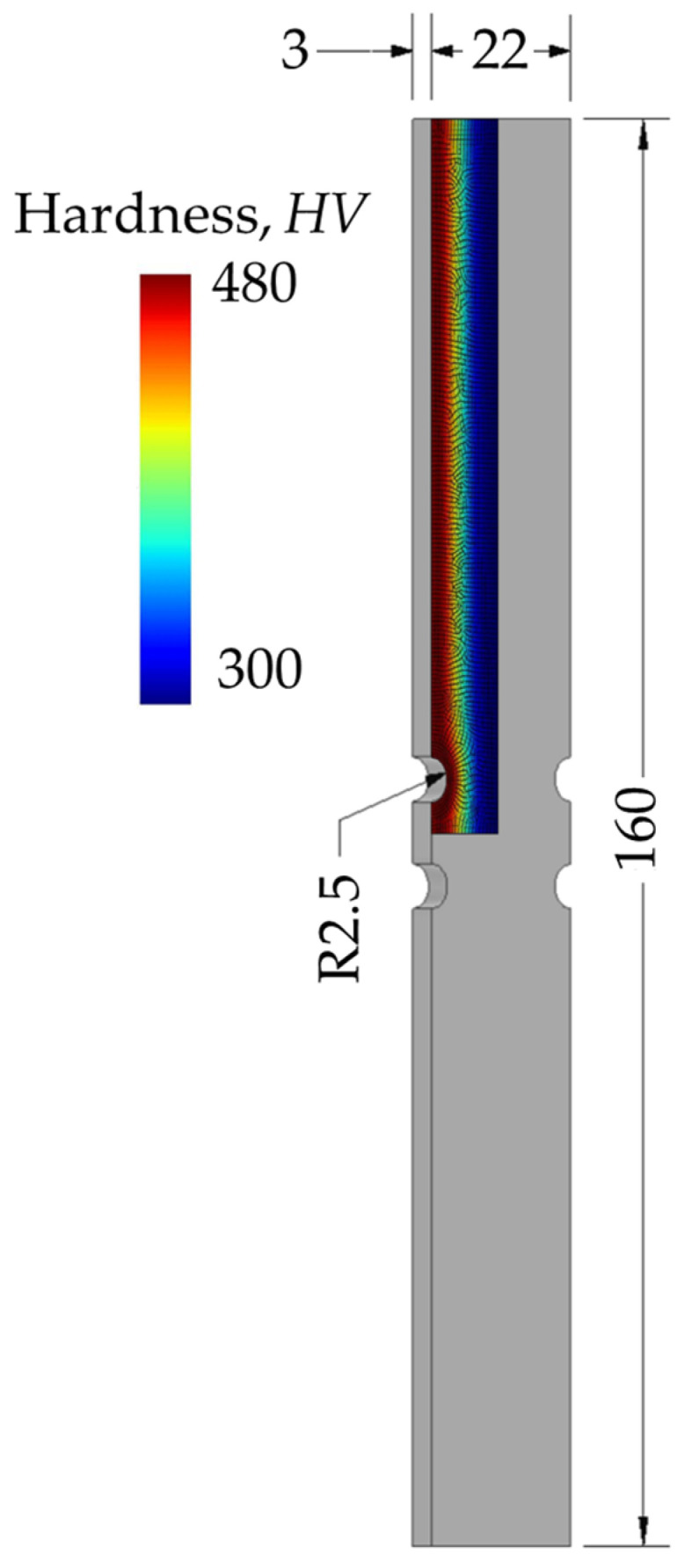
Dimensions and hardness distribution of one component from test dataset used as example.

To provide a more comprehensive analysis and presentation of results, this section presents one example from the test dataset involving a surface-hardened component with four opposite notches with a radius of 2.5 mm. The component has a total width of 22 mm, a length of 160 mm, and a thickness of 3 mm, with its geometry and hardness distribution illustrated in Figure 13.

**Figure 14:**
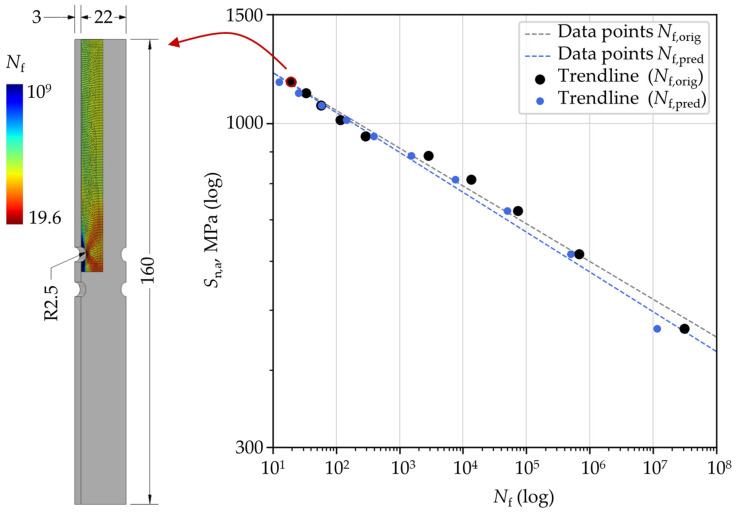
One example of an *S*-*N* component curve with FE-generated data and ANN-predicted data. For the data point marked with red, the distribution of the number of load cycles to failure as obtained from the FE analysis is shown.

The datapoints used to construct *S*-*N* curves for this component are presented in Figure 14, showing both the target (FE-based) fatigue life results and the corresponding fatigue life estimations from the ANN model. Trendlines are included to visually represent the overall fatigue behavior based on these data points. The net nominal stress amplitude *S*_n,a_ is calculated considering the net cross-section of the component obtained by excluding the stress concentrator from the gross cross-section. The figure also highlights the distribution of the number of load cycles to failure for one of the 10 loading conditions (marked in red) obtained through FEA. From this distribution, the minimum number of load cycles to failure is plotted on the *S*-*N* curve and used as input to the ANN for each loading condition. It is evident that the estimated *S*-*N* curve closely follows the original *S*-*N* curve, maintaining a similar slope. In this example, the majority of individual fatigue life estimations fall on the conservative side. The estimates in the low-cycle fatigue region show better alignment with the original values compared to those in the high-cycle fatigue region, consistent with the findings discussed previously.

Using the developed ANN model, it is possible to construct component *S*-*N* curves for various input parameters that fall within the predefined ranges the network was trained on. As demonstrated here, these curves can be generated with reasonable accuracy, avoiding the time-intensive finite element analysis and significantly speeding up the analysis process.

## 5. Conclusions

A parametrized computational model based on finite element analysis was developed for surface-hardened and through-hardened component-like specimens, providing the data needed to train an artificial neural network (ANN). Estimating the mechanical behavior of components for fatigue life prediction using the FE model can be time-consuming, particularly due to material nonlinearities. To address this, a surrogate ANN model was introduced to quickly and directly estimate the fatigue life of a component based on its geometry, material properties, and loading conditions. The FE simulations were performed on a wide range of geometry configurations, including various types and numbers of stress concentrators and different material states. To evaluate the generalization capability of the ANN, the model was tested on an independent dataset generated from a parameter space distinct from that used for training.

The ANN demonstrated satisfactory overall performance, with around 85% of the data points falling within the constructed range (a fatigue life factor of 3). When evaluated separately based on fatigue life ranges, low-cycle fatigue, and high-cycle fatigue (with the separation at 10^4^ cycles), the model showed higher accuracy in the LCF range, where more than 87% of predictions fell within the specified range (fatigue life factor 3) compared to 63% in the HCF range. This suggests that the ANN model performs more consistently in the LCF regime, while the HCF range exhibits greater variability in the predicted data points. Regarding different heat treatment types, almost 100% of the data points for through-hardened components fall within the range with a fatigue life factor of 3. For surface-hardened components, 79% of the data points fall within the range with a factor of 3, while more than 90% fall within the range with a factor of 5. These results indicate that while predicting the fatigue life of surface-hardened components is more challenging, the model still provides reasonably reliable estimates.

Additionally, it was shown that the developed ANN model enables the construction of component-specific *S*-*N* curves within the boundaries of the examined cases with reasonable accuracy, eliminating the need for finite element analysis and separate fatigue life calculations. While the model demonstrates strong agreement with the FE-based results, it is important to note that experimental fatigue data typically show greater scatter, which is not reflected in simulation-based datasets. Nonetheless, the presented approach serves as a valuable and efficient tool for preliminary fatigue assessment and can be further refined through integration with experimental results in future work.

## Figures and Tables

**Table 1:** Finite element model properties and descriptions.

Property	Description
Geometry	2D quarter model of thin plate with opposite notches or central hole/holes (parametrized dimensions)
Material model— surface-hardened material	20 multilinear isotropic hardening models defined with 20 stabilized cyclic stress–strain curves; details on applying pseudo-temperature method to implement these curves for surface-hardened material are provided in [23]
Material model— through-hardened material	Single multilinear isotropic hardening model defined by one stabilized cyclic stress–strain curve; expressions used to derive stress-strain curves from hardness values are provided in [26]
Element type	PLANE223 (quadratic 8-node element) for surface-hardened materials; PLANE183 (quadratic 8-node element) for through-hardened cases
Degrees of freedom	Displacement in *x* and *y*; non-physical temperature (when using PLANE223)
Mesh	Parametrized; 20 divisions around ¼ of stress concentrator circumference, denser towards area of stress concentrator
Boundary conditions	Symmetry conditions applied to quarter model; uniaxial loading condition
Analysis type	2D plane stress
Results	Stress and strain amplitudes at nodes, used for fatigue life calculations
Geometry	Parametrized model created using Ansys^®^ Mechanical APDL, Release 2023 R1, with pyMAPDL (version 0.64.0) integration for Python scripting

**Table 2:** Limits and parameter space of all input variables, separated into geometry and material variables, used to train ANN.

Geometry variables	Half-width, *W*/2	10, 12, 14 … 30 mm		
	Radius, *R*	2, 3, 4 … 12 mm		
	Number of stress concentrators, *N*	1, 2, 3 … 8		
	Types of stress concentrator	Opposite notches, central holes		
Material variables	Hardened notch	Surf.-hardened	8 combinations of surface and core hardness, *HV*_s_ and *HV*_c_ *	
	Non-hardened notch	Surf.-hardened	8 combinations of surface and core hardness, *HV*_s_ and *HV*_c_ *	
		Through-hardened	Hardness, *HV*	300, 370, 450, 550 HV

* Combinations of surface and core hardness (*HV*_s_, *HV*_c_): (630, 400); (630, 450); (600, 400); (580, 350); (580, 300); (550, 300); (500, 280); (500, 250).

**Table 3:** Limits and parameter space of all input variables, divided into geometry and material variables, used for testing ANN.

Geometry variables	Half-width, *W*/2	11, 13, 15 … 29 mm		
	Radius, *R*	2.5, 3.5, 4.5 … 11.5 mm		
	Number of stress concentrators, *N*	1, 2, 3 … 8		
	Types of stress concentrator	Opposite notches, central holes		
Material variables	Hardened notch	Surf.-hardened	8 combinations of surface and core hardness, *HV*_s_ and *HV*_c_ *	
	Non-hardened notch	Surf.-hardened	8 combinations of surface and core hardness, *HV*_s_ and *HV*_c_ *	
		Through-hardened	Hardness	325, 390, 460, 525 HV

* Combinations of surface and core hardness (*HV*_s_, *HV*_c_): (640, 360); (620, 415); (590, 320); (570, 290); (550, 320); (540, 330); (480, 300); (500, 270).

**Table 4:** A total of 3 of the 400 combinations of input variables used to generate the dataset to train the surrogate ANN model. All combinations are available in the Supplementary Material (Table S1).

Comb. Number	Num. of Notches/Holes, *N*	Radius, *R*, mm	Half-Width, *W*/2, mm	Stress Concentrator	Core Hardness, *HV*_c_, HV	Surface Hardness, *HV*_s_, HV	Hardened Notch	Half-Length, *L*/2, mm
1	4	7	20	Notch	300	580	No	80
2	5	9	20	Notch	250	500	Yes	122
3	8	4	28	Hole	250	500	No	116

**Table 5:** Sample training dataset for surrogate ANN model with 6 out of 4000 data entries presented. All other data entries are provided in the Supplementary Material (Table S3).

Combination Number	Loading Number	Input Variable	Output Variables from FEA		Output Variables for ANN	
		Gross Nominal Stress, *S*_g_, MPa	Maximum Strain Amplitude, *ε*_a,max_, mm/mm	Maximum Stress Amplitude, *σ*_a,max_, MPa	Number of Cycles to Failure, *N*_f_	Number of Cycles to Failure, log(*N*_f_)
1	1	290.13	0.0021	370.75	6.25 × 10^4^	4.80
1	… *	…				
1	10	728.79	0.0267	579.64	5.50 × 10^1^	1.74
2	1	226.40	0.0011	225.84	1.72 × 10^7^	7.24
2	…	…				
2	10	568.70	0.0296	854.28	1.62 × 10^1^	1.21
3	1	339.74	0.0018	342.03	1.03 × 10^5^	5.01
3	…	…				
3	10	853.39	0.0279	694.67	6.43 × 10^1^	1.81

* Load cases numbered 2 to 9 for each combination have been omitted from this table for clarity but are included in the Supplementary Material (Table S3).

**Table 6:** Final settings and parameters of surrogate artificial neural network.

Network Parameter/Setting	Value
Optimizer	Adam (Adaptive Moment Estimation), *α* = 0.001
Activation function	Leaky ReLu, *α* = 0.01 (hidden lay.) Purelin (output lay.)
Loss function	*MSE*
Batch size	32
Number of hidden layers	1
Number of neurons per hidden layer	Determined by growth method (23)
Epochs	300
Early stopping—patience	10
Regularization	L2 (*λ* = 0.001)

**Table 7:** Performance metrics on training and test datasets, as well as on test datasets separated into LCF range, HCF range, through-hardened components, and surface-hardened components.

Metric	Train Dataset	Test Dataset	LCF Range (Test Set)	HCF Range (Test Set)	Through-Hardened (Test Set)	Surface-Hardened (Test Set)
*R* ^2^	0.952	0.935	0.833	0.734	0.975	0.924
*MAE*	0.201	0.255	0.230	0.332	0.153	0.293
*MAPE*, %	7.618	9.739	10.746	6.664	5.582	11.315
*MSE*	0.085	0.129	0.101	0.214	0.039	0.162
*RMSE*	0.291	0.359	0.317	0.462	0.198	0.403

## Data Availability

Detailed descriptions of the properties of the finite element model used for the generation of the training data as well as detailed neural network architecture are provided throughout the paper. The data used to train and test the model are provided in the Supplementary Material. Further inquiries can be directed to the corresponding author.

## Supplementary Materials

The following supporting information can be downloaded at: https://www.mdpi.com/article/10.3390/ma18122756/s1, Table S1: All components used for training the artificial neural network model; Table S2: All components used for testing the artificial neural network model; Table S3: FEA generated input data for training the artificial neural network model; Table S4: FEA generated input data for testing the artificial neural network model.
